# Intensive care admission in patients with hellp syndrome in a tertiary referral hospital

**DOI:** 10.1186/2197-425X-3-S1-A909

**Published:** 2015-10-01

**Authors:** E Gedik, N Yücel, T Sahin, E Koca, YZ Çolak, T Togal

**Affiliations:** Baskent University, Ankara, Turkey; Inonu University, Malatya, Turkey; Ersin Arslan State Hospital, Gaziantep, Turkey; Malatya State Hospital, Malatya, Turkey

## Introduction

Every year, 500.000 mothers die from pregnancy-related complications, and 99% of these deaths occur in low- and middle-income countries [1]. These maternal deaths occur from complications associated with pre-eclampsia or eclampsia; hemolysis elevated liver enzymes, and low platelet (HELLP) syndrome, or other hypertensive disorder of pregnancy [2].

## Objectives

To identify risk factors affecting maternal outcome among women with Hemolysis, Elevated Liver Enzymes, and Low Platelets (HELLP) syndrome who required transfer for critical care.

## Methods

All 77 women with HELLP syndrome who sought care for delivery and postpartum assessment at the emergency department, and who were treated in our intensive care unit between January 2007 and July 2012 were identified, retrospectively. Findings were analyzed according to surviving and non-surviving patients.

## Results

Maternal mortality rate was 14% and perinatal death occurred in 24 of 81 fetuses and newborn (30%). The most common cause of maternal complications was disseminated intravascular coagulation in 22 patients (29%), acute renal failure in 19 patients (25%), and postpartum hemorrhage in 16 patients (21%). Compared with surviving women, non-surviving women had higher mean values for international normalized ratio (*p* < 0.0001), levels of serum aspartate aminotransferase (*p* < 0.0001), serum alanine aminotransferase (*p* < 0.0001), lactate dehydrogenase *(p <* 0.0001), and bilirubin (*p* = 0.040) and lower platelet count (*p* = 0.005).

## Conclusions

The patients with HELLP syndrome should be treated in intensive care unit, especially after cesarean delivery. Disseminated intravascular coagulation is major risk factor affecting maternal outcome.
